# Diffusion tensor imaging along the perivascular space and choroid plexus volume for assessing the brain glymphatic system in overt hypothyroidism

**DOI:** 10.3389/fneur.2026.1747261

**Published:** 2026-04-10

**Authors:** Hong Yu, Yuting Wang, Feifei Zheng, Hailin Shao, Qing He, Xuehuan Liu, Hao Wang, Xiao Gao, Zuoxi Li, Jun Liu

**Affiliations:** 1 Department of Radiology, The Fourth Central Hospital Affiliated to Tianjin Medical University, Tianjin, China; 2 Department of Radiology, Tianjin Union Medical Center, The First Affiliated Hospital of Nankai University, Nankai University, Tianjin, China; 3 Department of Endocrinology and Metabolism, Tianjin Medical University General Hospital, Tianjin, China

**Keywords:** cerebrospinal fluid, choroid plexus volume, diffusion tensor imaging along the perivascular space, glymphatic system, hypothyroidism

## Abstract

**Introduction:**

The neurocognitive impairment in overt hypothyroidism (OH) is well-recognized, but its underlying mechanisms remain unclear. The glymphatic system, a recently discovered brain waste-clearance pathway, represents a promising yet unexplored target in this context. This study aimed to non-invasively assess glymphatic function in OH patients using the DTI-ALPS index and choroid plexus (CP) volume, and to investigate their relationships with thyroid function and cognitive performance.

**Methods:**

We enrolled 40 patients with overt hypothyroidism (OH) and 42 healthy controls. All participants underwent 3D-T1 weighted imaging (3D-T1WI) and diffusion tensor imaging (DTI). Glymphatic activity was assessed using the DTI-ALPS index, and choroid plexus (CP) volume was segmented from 3D-T1WI images. After adjusting for covariates, correlations between these imaging markers and thyroid hormones as well as neuropsychological scale scores were analyzed.

**Results:**

After confounder adjustment, OH patients demonstrated a significantly reduced DTI-ALPS index and enlarged CP volume compared to controls. Critically, a lower DTI-ALPS index was associated with a larger CP volume. Serum FT3 levels and MoCA scores showed strong positive correlations with the DTI-ALPS index but negative correlations with CP volume.

**Discussion:**

Our study provides the first direct evidence of glymphatic system dysfunction in OH, linking it to thyroid hormone levels and cognitive deficits. The DTI-ALPS index and CP volume emerge as novel and promising neuroimaging biomarkers, offering new insights into the pathophysiology of hypothyroidism-related neurocognitive decline and potential targets for future interventions.

## 1 Introduction

Hypothyroidism is a systemic hypometabolic syndrome caused by reduced production or impaired action of thyroid hormones due to various etiologies. Primary hypothyroidism accounts for approximately 99% of all cases. Thyroid hormones are essential for normal brain development and play an important role in maintaining neural homeostasis in adulthood (1). Hypothyroidism can lead to a wide range of neuropsychiatric manifestations, including apathy, depression, cognitive impairment, mood disturbances, and even psychosis (2, 3). Previous studies have demonstrated that structural and functional abnormalities in specific brain regions of patients with hypothyroidism are closely associated with their neuropsychiatric symptoms (4, 5). Previous neuroimaging studies further suggest that patients with hypothyroidism exhibit regional gray matter volume abnormalities (6, 7), accompanied by white matter microstructural damage (5, 8). Functionally, resting-state MRI studies have shown altered local spontaneous brain activity and disrupted functional connectivity in hypothyroidism, involving large-scale networks such as the default mode network, frontoparietal control network, and emotion-related brain regions (9). Moreover, accumulating evidence suggests that hypothyroidism is associated with cognitive decline and the development and progression of neurodegenerative diseases, particularly Alzheimer’s disease (AD) (1, 10, 11). However, the biological mechanisms linking hypothyroidism to cognitive decline and neurodegeneration remain poorly understood.

Impaired clearance of metabolic waste from the brain has been considered an important pathological component of cognitive decline and neurodegenerative diseases such as AD. Cerebrospinal fluid (CSF) is primarily produced by the choroid plexus (CP) and circulates through the ventricular system, exiting the brain via the median aperture (foramen of Magendie) and the lateral apertures (foramina of Luschka) of the fourth ventricle. The recently proposed glymphatic system hypothesis emphasizes that the glymphatic pathway facilitates the clearance of toxic metabolic products from the central nervous system through the exchange of CSF along periarterial spaces and interstitial fluid in the brain parenchyma (12). This process is regulated by multiple factors, including arterial pulsatility, sleep–wake state, astroglial water transport mechanisms, and CSF production and circulation dynamics. Prior studies have also suggested that cognitive decline and AD may be related to glymphatic dysfunction (13, 14). In addition, thyroid hormones can enter the CSF via transmembrane transporters or through transthyretin secreted by the CP (15). Glymphatic transport relies on astrocytic endfoot AQP4 to support CSF–interstitial fluid exchange (16). Experimental data indicate that T3 can modulate AQP4 expression/polarization, suggesting a plausible pathway by which thyroid hormone deficiency may impair perivascular exchange and glymphatic function (17). Accordingly, if hypothyroidism alters CSF dynamics or perivascular exchange, it may impair brain waste clearance, thereby providing a more targeted mechanistic framework for its neuropsychiatric manifestations and increased risk of cognitive decline. Given reports linking hypothyroidism to reversible dementia and the pathological progression of AD, and the growing recognition of glymphatic dysfunction as an important contributor to cognitive disorders including AD (18–20), we hypothesize that glymphatic impairment may represent a potential pathway connecting hypothyroidism to cognitive abnormalities.

At present, gadolinium-enhanced magnetic resonance imaging (MRI) is considered the gold standard for assessing glymphatic function; however, its clinical application is limited by the invasiveness of contrast agent administration (21, 22). Taoka and colleagues developed a diffusion tensor imaging (DTI) along the perivascular space (ALPS) index, which enables noninvasive evaluation of glymphatic function (23). This method quantifies the diffusion characteristics of water molecules surrounding the deep medullary veins and has been subsequently validated by dynamic contrast-enhanced imaging to correlate with glymphatic clearance function (24). Numerous researchers have since employed this technique to investigate glymphatic system function in neurodegenerative diseases, including Alzheimer’s disease, Parkinson’s disease, and other forms of dementia (25, 26). However, the relationship between the DTI-ALPS index and hypothyroidism has not yet been systematically investigated. Within glymphatic circulation, the CP is a key structure for CSF production, and its structural phenotype may reflect CSF-production–related status and CP tissue changes. Evidence suggests that changes in CP volume may be related to glymphatic function (27, 28). Moreover, CP volume has been reported to correlate with disease severity in frontotemporal dementia and AD, highlighting its potential as an imaging biomarker for glymphatic-related alterations (29, 30).

Although the DTI-ALPS index and CP volume may characterize different components of glymphatic circulation, evidence integrating these two measures in overt hypothyroidism (OH) remains limited. Whether OH is associated with impaired glymphatic function and how such imaging markers relate to thyroid function indices and neuropsychological performance have not been systematically reported. We hypothesized that patients with OH exhibit glymphatic system dysfunction, manifested by alterations in the DTI-ALPS index and CP volume. Therefore, the present study aimed to investigate glymphatic system alterations in patients with OH, with the following objectives: (1) to assess glymphatic-related imaging phenotypes in patients with OH using the DTI-ALPS index and CP volume; and (2) to explore the associations of the DTI-ALPS index with thyroid hormone levels, thyroid-related antibodies, and neuropsychological function.

## 2 Materials and methods

### 2.1 Participants

This study recruited newly diagnosed, treatment-naive adults with primary OH between July 2024 and July 2025. Eligible patients underwent brain MRI if they met the following criteria: (1) right-handed; (2) at least 6 years of formal education; (3) aged 18–60 years; (4) diagnosed with OH according to the “Guidelines for the Diagnosis and Treatment of Adult Hypothyroidism (2017 edition)”; and (5) complete clinical data available. Exclusion criteria included a history of neurological or psychiatric disorders, structural lesions of the central nervous system, severe white matter hyperintensities or (early) confluent lesions (Fazekas score ≥ 2), prior endocrine disorders or non-thyroid autoimmune diseases, cardiovascular or other major chronic illnesses, significant visual or auditory impairments, history of substance abuse, pregnancy or lactation, and any contraindications to MRI. A total of 40 eligible patients with OH were included. Additionally, 42 healthy controls (HCs) with normal thyroid function were recruited. This study was approved by the Institutional Review Board of Tianjin Fourth Central Hospital (Approval No. SZXLL-2023-KY045) and was conducted in accordance with the ethical principles of the Declaration of Helsinki (2013). Written informed consent was obtained from all participants.

### 2.2 Clinical data collection and neuropsychological assessment

All participants underwent comprehensive clinical and neuropsychological evaluations. Basic demographic information, including age, sex, and years of education, was recorded. Venous blood samples were collected from all participants after a 10-h overnight fast at 8:00 a.m., and serum levels of thyroid-stimulating hormone (TSH), free triiodothyronine (FT3), and free thyroxine (FT4) were measured using magnetic particle chemiluminescence immunoassay. Cognitive function was assessed using the Beijing version of the Montreal Cognitive Assessment (MoCA), which evaluates multiple cognitive domains, including visuospatial/executive function, naming, memory and delayed recall, attention, language, abstraction, and orientation. Emotional status was assessed using the Hamilton Depression Rating Scale-24 (HAMD-24) and the Hamilton Anxiety Rating Scale (HAMA). All neuropsychological assessments were conducted by trained clinicians under the supervision of senior neurologists.

### 2.3 MRI data acquisition

Brain MRI scans were performed using a 3.0 T MRI scanner (SIGNA Architect, GE, USA) equipped with a 20-channel head and neck coil. During scanning, participants were instructed to keep their eyes closed, remain relaxed, but stay awake. Diffusion MRI data were acquired using a single-shot echo-planar imaging sequence with a multi-b-value scheme. The acquisition parameters were as follows: echo time (TE) = 85 ms; repetition time (TR) = 8,000 ms; slice thickness = 1 mm; acquisition matrix = 224 × 224; field of view (FOV) = 224 × 224 mm^2^; flip angle = 90°; diffusion weighting (b value) = 1,000 s/mm^2^ with 32 diffusion directions, and a single b = 0 s/mm^2^ image (b0). The total acquisition time was 4 min and 48 s. Routine MRI sequences, including axial T1-weighted imaging (T1WI), T2-weighted imaging (T2WI), and T2-FLAIR, were performed to exclude structural abnormalities. High-resolution three-dimensional T1-weighted imaging (3D T1) was acquired using a magnetization-prepared rapid gradient-echo sequence with the following parameters: TE = 3.1 ms; TR = 7.7 ms; slice thickness = 1 mm; acquisition matrix = 256 × 256; FOV = 256 × 256 mm^2^; flip angle = 8°. The acquisition time was 5 min and 14 s.

### 2.4 Diffusion image processing

**Figure 1:**
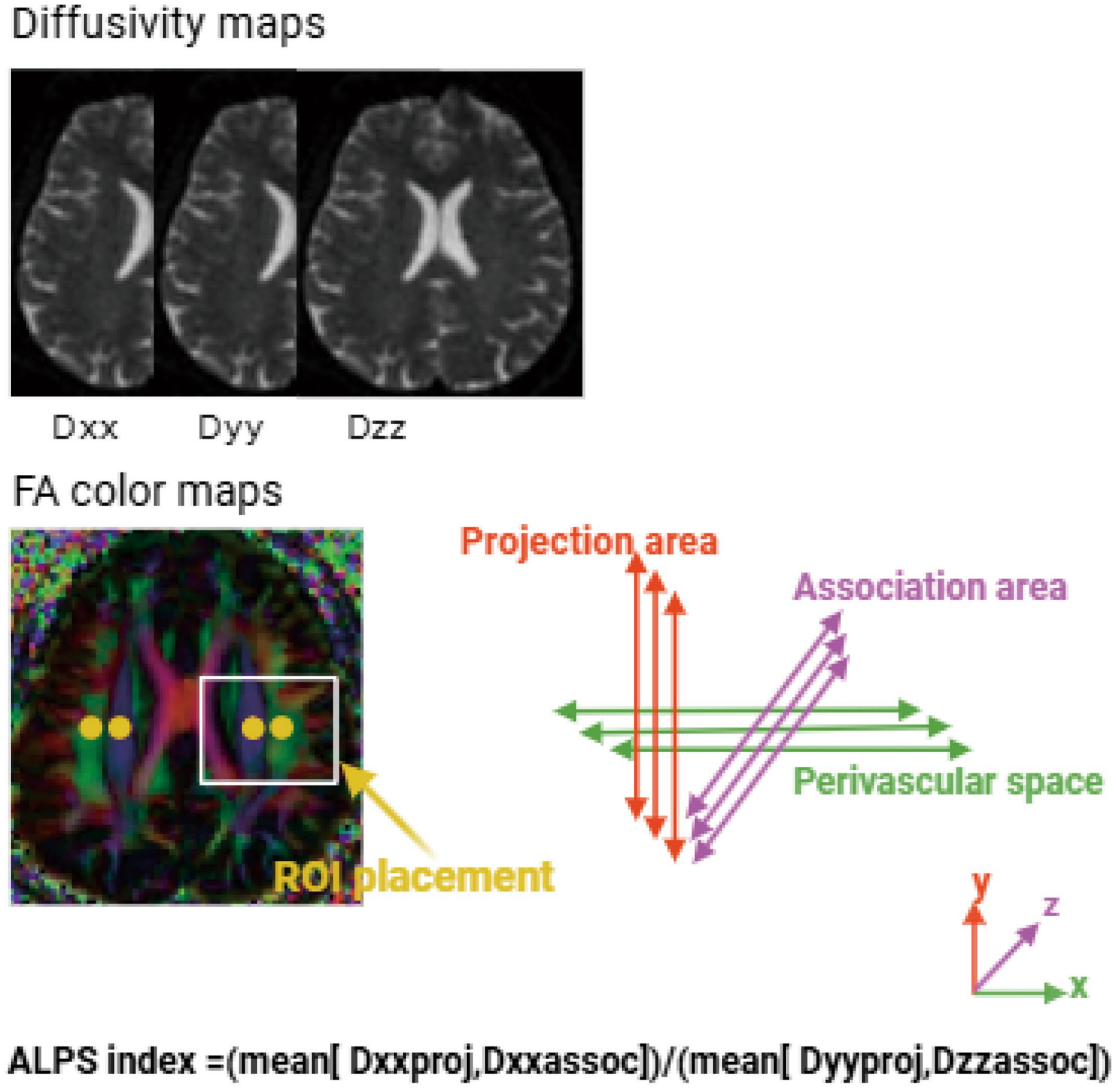
ALPS index calculation workflow. DTI images were processed to obtain color-coded FA maps and tensor images. Circular regions of interest (ROIs) with a diameter of 6 mm were placed in the projection and association fiber areas identified on the color-coded FA map. The MNI coordinates of the ROI centers in the projection-fiber region were (24, −12, 24) for the right hemisphere and (−28, −12, 24) for the left hemisphere. The MNI coordinates of the ROI centers in the association-fiber region were (36, −12, 24) for the right hemisphere and (−40, −12, 24) for the left hemisphere. The diagonal elements of the diffusion tensor (Dxx, Dyy, Dzz), representing anisotropic water diffusion along the *X*, *Y*, and *Z* directions, were extracted from the tensor images. The schematic simultaneously shows the orientation of the perivascular space relative to the fiber directions, indicating that the perivascular space is perpendicular to both the projection and association fibers.

DTI data were processed using DSI Studio (version 2024). Raw diffusion datasets were stored in DICOM format and underwent visual quality assessment. A brain mask was applied for skull stripping to remove non-brain tissue. Preprocessing further included correction of Gibbs ringing artifacts, Rician noise bias correction, and correction for head motion and eddy-current–induced distortions. After preprocessing, individual color-coded fractional anisotropy (FA) maps were generated. ROIs for ALPS calculation were placed on the axial color-coded FA map at the level of the body of the lateral ventricle, following the procedure described by Taoka et al. (23) and subsequent studies (31). Two circular regions of interest (ROIs; 6 mm per side) were placed in each hemisphere in each hemisphere within the projection-fiber region (corona radiata; predominant superior–inferior orientation) and the association-fiber region (superior longitudinal fasciculus; predominant anterior–posterior orientation), avoiding the ventricular boundary and CSF signal to minimize partial-volume effects (24). Specifically, ROIs were positioned on axial slices at the level of the body of the lateral ventricle within the periventricular projection-fiber (corona radiata) and association-fiber (superior longitudinal fasciculus) territories. Each participant’s FA map was nonlinearly registered to the MNI standard space using ANTs,^1^ and the inverse transformations were subsequently applied to warp the ROIs back into each participant’s native space (Figure 1). All warped ROIs were visually quality-checked on individual color-coded FA maps. ROIs were visually inspected to ensure appropriate alignment with the target white-matter tracts and to minimize CSF contamination/partial-volume effects. ROIs with clear mislocalization were excluded from sampling, whereas ROIs with minor misalignment were minimally refined within a small range while keeping ROI size and shape unchanged. Following ROI placement, diffusion tensor directional diffusivities along the *x*-, *y*-, and *z*-axes were extracted within each ROI using FSL 6.0.6^2^ and denoted as Dxx, Dyy, and Dzz, respectively. The DTI-ALPS index was calculated according to the method described by Taoka et al. (23). For each hemisphere, the ALPS index was defined as the mean x-axis diffusivity in the projection-fiber ROI (Dxx_proj) and association-fiber ROI (Dxx_asso) divided by the mean of the y-axis diffusivity in the projection-fiber ROI (Dyy_proj) and the z-axis diffusivity in the association-fiber ROI (Dzz_asso). The numerator reflects relatively increased diffusivity along the presumed perivascular space (PVS) direction (*x*-axis), whereas the denominator serves as a reference based on orthogonal directions (*y*- and *z*-axes). The left and right ALPS index were averaged for subsequent statistical analyses, with lower ALPS values indicating poorer glymphatic-related function.

### 2.5 Choroid plexus volume segmentation

Three-dimensional T1-weighted images were used to calculate the CP volume and intracranial volume (ICV) for each participant. Brain structural processing and automatic segmentation were performed using FastSurfer,^3^ which includes bias field correction, skull stripping to remove non-brain tissue, delineation of bilateral cortical gray–white matter boundaries, and segmentation of brain tissue into white matter, gray matter, and CSF (32). Subsequently, two radiologists performed quality control of CP and ICV measurements for all 82 participants. The final automated segmentation results were reviewed and approved by a neuroimaging researcher, and no manual correction was required. To reduce inter-individual variability, CP volumes were normalized according to previous studies by expressing them as the ratio of CP volume to ICV multiplied by 1,000 (30).

### 2.6 Statistical analysis

Categorical variables were compared using the chi-square test and are presented as counts and percentages. Continuous variables with a normal distribution were compared using the Student’s t-test and are presented as mean ± standard deviation (SD), whereas non-normally distributed continuous variables were compared using the Mann–Whitney U test and are presented as median and interquartile range (IQR). Analysis of covariance (ANCOVA) was used to compare DTI-ALPS index and CP volumes between the OH and healthy control (HC) groups, and partial correlation analysis was applied to assess the associations between DTI-ALPS index and CP volumes. Further partial correlation analyses were conducted to examine the relationships of disease duration, thyroid hormone levels, and neuropsychological scores with ALPS index and CP volumes, controlling for potential confounders. All ANCOVA and partial correlation analyses were adjusted for age, sex, body mass index (BMI), smoking, alcohol consumption, and years of education. Statistical analyses were performed using R software (version 3.4.3) and IBM SPSS Statistics (version 25.0), with two-tailed tests and a significance level set at *p* < 0.05. Multiple comparisons were corrected using the false discovery rate (FDR) method.

## 3 Results

### 3.1 Demographic, clinical, and neuropsychological characteristics

A total of 82 participants were included in the present study, comprising 40 patients with overt hypothyroidism (OH; 23 females and 17 males; mean age, 47.63 ± 1.74 years) and 42 healthy controls (HCs; 25 females and 17 males; mean age, 43.55 ± 1.52 years). The two groups were well matched in demographic and lifestyle characteristics, with no significant between-group differences in age, sex, body mass index (BMI), years of education, smoking status, or alcohol consumption (all *p* > 0.05). Compared with the HC group, patients with OH exhibited significantly lower Montreal Cognitive Assessment (MoCA) scores and higher Hamilton Depression Rating Scale (HAMD) and Hamilton Anxiety Rating Scale (HAMA) scores (P_FDR < 0.05) (Table 1). Overall, these results indicate poorer cognitive performance and greater anxiety symptom burden in the OH group.

**Table 1:** Demographic characteristics, clinical variables, and neuropsychological assessments.

Variables	OH (*n* = 40)	HC (*n* = 42)	*χ^2^*/*t*/*z* value	*p*value
Demographic				
Age (years)	47.63 ± 10.21	43.55 ± 10.54	1.767	0.081
Sex [*n* (%)]			0.035	0.852
Female	23 (57.5%)	25 (59.5%)		
Male	17 (42.5%)	17 (40.5%)		
BMI (kg/m^2^)	23.54 ± 0.29	23.98 ± 0.44	−0.831	0.408
Education (years)	12 (9, 16)	12 (9, 16)		
Smoking status			0.732	0.392
Never smoking	23 (57.5%)	27 (64.3%)		
Former smoking	7 (17.5%)	2 (4.8%)		
Current smoking	10 (25.0%)	13 (31.0%)		
Drinking			0.463	0.496
Never drinking	23 (57.5%)	21 (50.0%)		
Ever/current drinking	17 (42.5%)	21 (50.0%)		
Clinical characteristic				
Duration (months)	8.93 ± 0.85			
FT3 (pmol/L)	3.12 (2.51, 3.28)	5.13 (4.35, 5.65)	−7.497	<0.001
FT4 (pmol/L)	8.88 (8.22, 9.35)	14.95 (12.4, 18.23)	−7.705	<0.001
TSH (mIU/L)	63.78 (45.24, 100.00)	2.15 (1.21, 3.16)	−7.802	<0.001
TPOAb (IU/mL)	3357.10 ± 158.02			
TGAb (IU/mL)	3673.53 ± 151.17			
Neuropsychological tests				
MoCA	26.5 (25, 28)	27 (26, 28)	−2.657	0.008
HAMD	4 (2, 8)	2 (0, 4)	−3.195	0.001
HAMA	14 (7.5, 17.5)	1 (1, 2)	−7.020	<0.001

HC, healthy controls; OH, overt hypothyroidism; BMI, Body Mass Index; TSH, thyroid-stimulating hormone; FT3, free triiodothyronine; FT4, free thyroxine; TgAb, anti-thyroglobulin antibodies; TPOAb, thyroid peroxidase antibodies. Bold indicates statistical significance; MoCA, Montreal Cognitive Assessment; HAMD-24, Hamilton Rating Scale for Depression-24; HAMA, Hamilton Anxiety Rating Scale. Bold indicates statistical significance.

### 3.2 Differences in DTI-ALPS index between patients with OH and HCs

**Figure 2:**
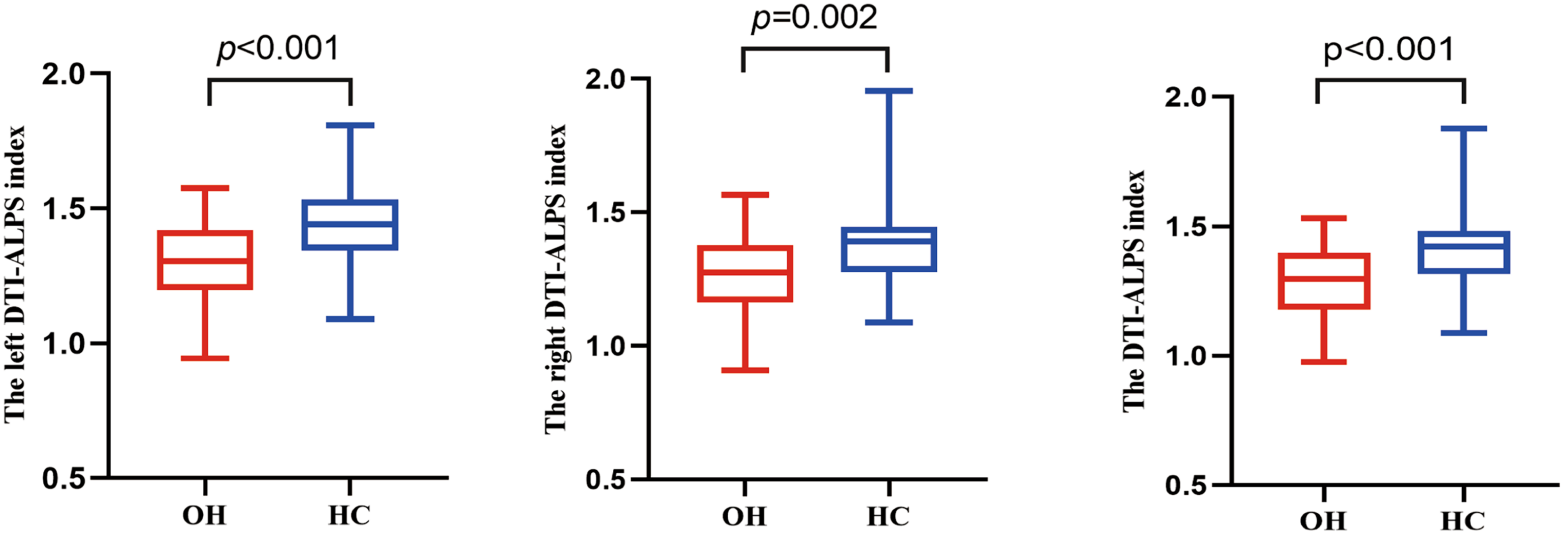
Box plots of DTI-ALPS index between OH and HC groups. The *y*-axis displays ALPS index values, while the *x*-axis categorizes the groups. Analysis of covariance (ANCOVA) was performed to compare ALPS index between OH and HC groups, with covariates including age, years of education, sex, body mass index, smoking, and drinking. HC, healthy control; OH, overt hypothyroidism; DTI-ALPS, diffusion tensor imaging (DTI) along the perivascular space (ALPS) index.

After adjusting for age, sex, BMI, years of education, smoking, and alcohol consumption, significant group differences in the DTI-ALPS index were observed between the OH and HC groups in the left hemisphere, right hemisphere, and whole-brain mean values (Table 2 and Figure 2). Specifically, patients with OH showed significantly lower DTI-ALPS index across all measures compared with HCs (P_FDR_ < 0.05). Overall, these findings indicate a consistent bilateral reduction in the DTI-ALPS index in OH.

**Table 2:** Comparison of DTI-ALPS index, CP volume, and PVS between OH and HC.

Variables	OH (*n* = 40)	HC (*n* = 42)	*χ^2^*/*t*/*z* value	*p* value	*P_FDR_*
The left DTI-ALPS index	1.31 ± 0.03	1.45 ± 0.03	−4.086	<0.001	<0.001
The right DTI-ALPS index	1.27 ± 0.02	1.40 ± 0.03	−3.367	0.001	0.002
The DTI-ALPS index	1.29 ± 0.02	1.42 ± 0.02	−4.048	<0.001	<0.001
The left CP volume (ratio of ICV × 10^3^)	0.91 ± 0.04	0.74 ± 0.03	3.457	0.001	0.002
The right CP volume (ratio of ICV × 10^3^)	0.88 ± 0.04	0.73 ± 0.02	3.406	0.001	0.002
The CP volume (ratio of ICV × 10^3^)	0.90 ± 0.04	0.73 ± 0.03	3.657	<0.001	<0.001

The one-way analysis of covariance (ANCOVA) was performed to control for the confounding effects of age, sex, body mass index, years of education, smoking, and drinking. CP volume = CP volume/ICV *1000; PVS volume fraction = PVS volume/(gray + white matter volume) *100%. HC, healthy control; OH, overt hypothyroidism; DTI-ALPS, diffusion tensor imaging (DTI) along the perivascular space (ALPS) index; CP, choroid plexus; PVS, perivascular space; ICV, Intracranial Volume; WM, white matter; SubCor, Subcortical Structures.

### 3.3 Differences of choroid plexus volume between patients with OH and HCs

**Figure 3:**
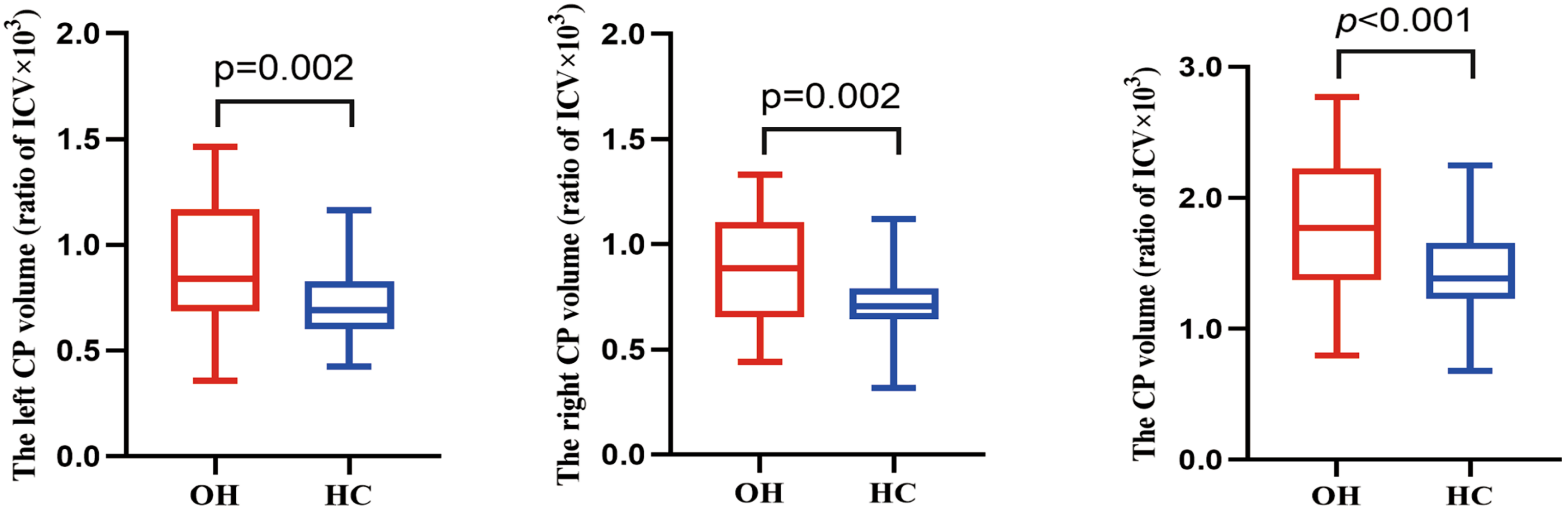
Box plots of CP volume between OH and HC groups. The *y*-axis displays CP volume, while the *x*-axis categorizes the groups. Analysis of covariance (ANCOVA) was performed to compare CP volume between OH and HC groups, with covariates including age, years of education, sex, body mass index, smoking, and drinking. HC, healthy control; OH, overt hypothyroidism; CP, choroid plexus.

As shown in Table 2 and Figure 3, significant differences in absolute CP volume were observed between the OH and HC groups in the left hemisphere, right hemisphere, and whole brain. After adjusting for age, sex, BMI, years of education, smoking, and alcohol consumption, patients with OH exhibited significantly larger absolute CP volumes in all regions compared with HCs (P_FDR_ < 0.05). Taken together, OH was characterized by a consistent bilateral increase in CP volume, suggesting a global alteration in CP-related structural phenotype.

### 3.4 Correlation between DTI-ALPS index and choroid plexus volume

**Figure 4:**
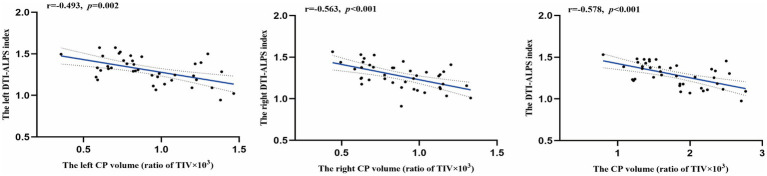
Relationships between choroid plexus volume and diffusion tensor imaging (DTI) along the perivascular space (ALPS) index. Correlation coefficients (*r*) and *p* values were obtained using partial correlation analysis, controlling for age, sex, body mass index, years of education, smoking status, and alcohol consumption. Trend lines depict linear regression with 95% confidence intervals (shaded). DTI-ALPS, diffusion tensor imaging (DTI) along the perivascular space (ALPS) index; CP, choroid plexus.

As shown in Figure 4, after adjusting for age, sex, BMI, years of education, smoking, and alcohol consumption, the DTI-ALPS index in the left hemisphere, right hemisphere, and whole brain showed significant negative correlations with absolute choroid plexus volume (*r* = −0.493, *p* = 0.002; *r* = −0.563, *p* < 0.001; *r* = −0.578, *p* < 0.001).

### 3.5 Correlations of the DTI-ALPS index with clinical variables and neuropsychological assessments

**Figure 5:**
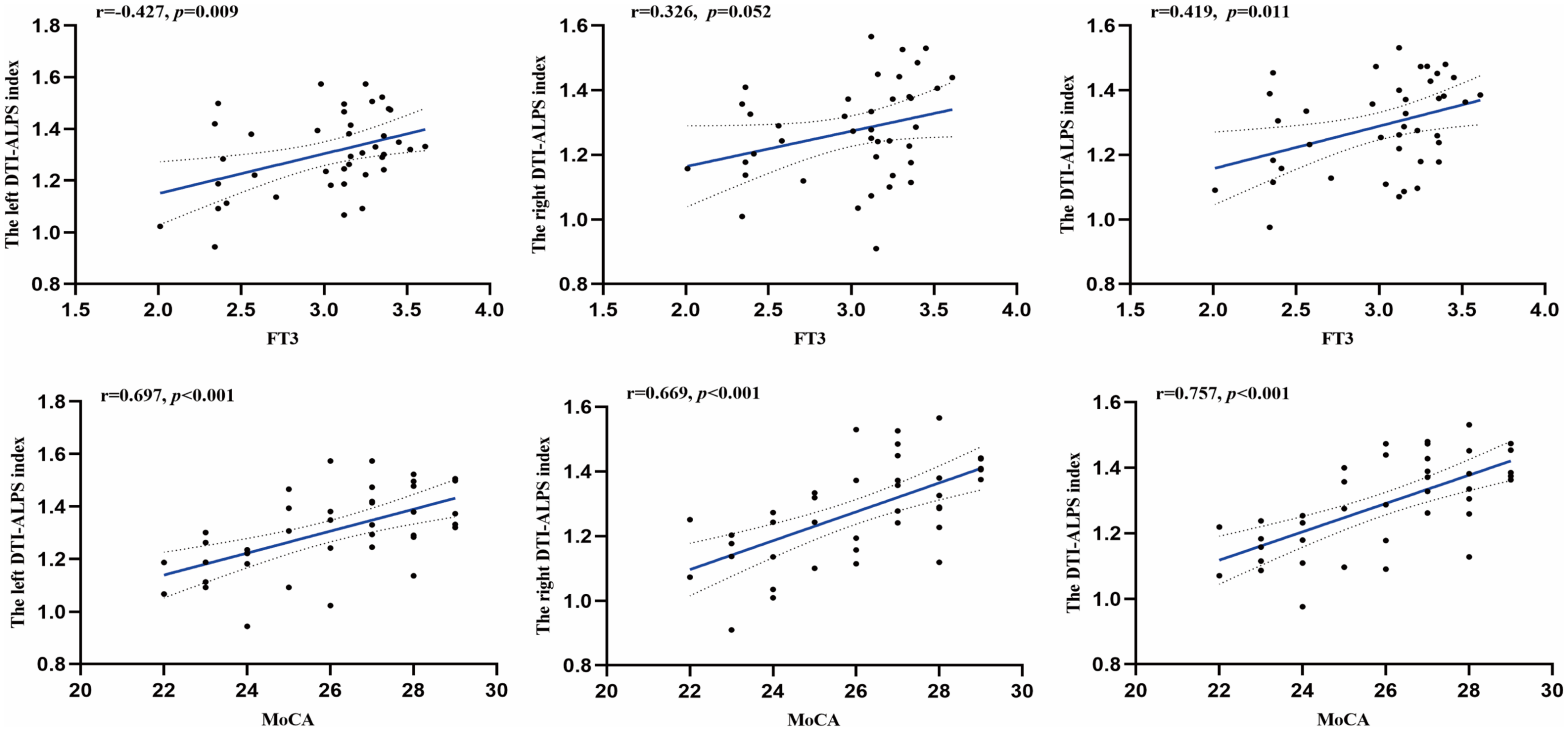
Relationships between DTI-ALPS index and clinical variables as well as neuropsychological assessments. Correlation coefficients (*r*) and *p* values were obtained using partial correlation analysis, controlling for age, sex, body mass index, years of education, smoking status, and alcohol consumption. Trend lines depict linear regression with 95% confidence intervals (shaded). FT3, free triiodothyronine; MoCA, Montreal Cognitive Assessment; DTI-ALPS, diffusion tensor imaging (DTI) along the perivascular space (ALPS) index.

As shown in Table 3 and Figure 5, after adjusting for age, sex, BMI, years of education, smoking, and alcohol consumption, no significant correlation was observed between disease duration and the DTI-ALPS index. FT3 levels were positively correlated with the left hemisphere and whole-brain mean DTI-ALPS index (*r* = 0.427, *p* = 0.009; *r* = 0.419, *p* = 0.011). Moreover, MoCA scores were positively correlated with the DTI-ALPS index of the left hemisphere, right hemisphere, and whole brain (*r* = 0.697, *p* < 0.001; *r* = 0.669, *p* < 0.001; *r* = 0.757, *p* < 0.001). Overall, the DTI-ALPS index showed consistent positive associations with FT3 levels and cognitive performance, whereas no detectable relationship with disease duration was observed after covariate adjustment.

**Table 3:** Associations between clinical characteristics, neuropsychological assessments, and DTI-ALPS index.

Variables	The left DTI-ALPS index		The right DTI-ALPS index		The DTI-ALPS index	
	*r*	*p* value	*r*	*p* value	*r*	*p* value
Duration (months)	−0.207	0.226	−0.242	0.155	−0.248	0.145
FT3 (pmol/L)	0.427	0.009	0.326	0.052	0.419	0.011
FT4 (pmol/L)	0.195	0.255	0.246	0.148	0.243	0.153
TSH (mIU/L)	0.040	0.815	−0.053	0.758	−0.005	0.977
TPOAb (IU/mL)	0.327	0.052	0.193	0.259	0.291	0.085
TgAb (IU/mL)	0.178	0.299	0.304	0.071	0.264	0.119
MoCA	0.697	<0.001	0.669	<0.001	0.757	<0.001
HAMD	−0.127	0.462	−0.041	0.813	−0.095	0.583
HAMA	0.185	0.280	0.244	0.152	0.236	0.165

Partial correlation analysis was performed to control for the confounding effects of age, sex, body mass index, years of education, smoking, and drinking. Trend lines depict linear regression with 95% confidence intervals (shaded). DTI-ALPS, diffusion tensor imaging (DTI) along the perivascular space (ALPS) index; FT3, free triiodothyronine; FT4, free thyroxine; TgAb, anti-thyroglobulin antibodies; TPOAb, thyroid peroxidase antibodies. Bold indicates statistical significance; MoCA, Montreal Cognitive Assessment; HAMD-24, Hamilton Rating Scale for Depression-24; HAMA, Hamilton Anxiety Rating Scale.

### 3.6 Correlations between CP volume, clinical variables, and neuropsychological measures

**Figure 6:**
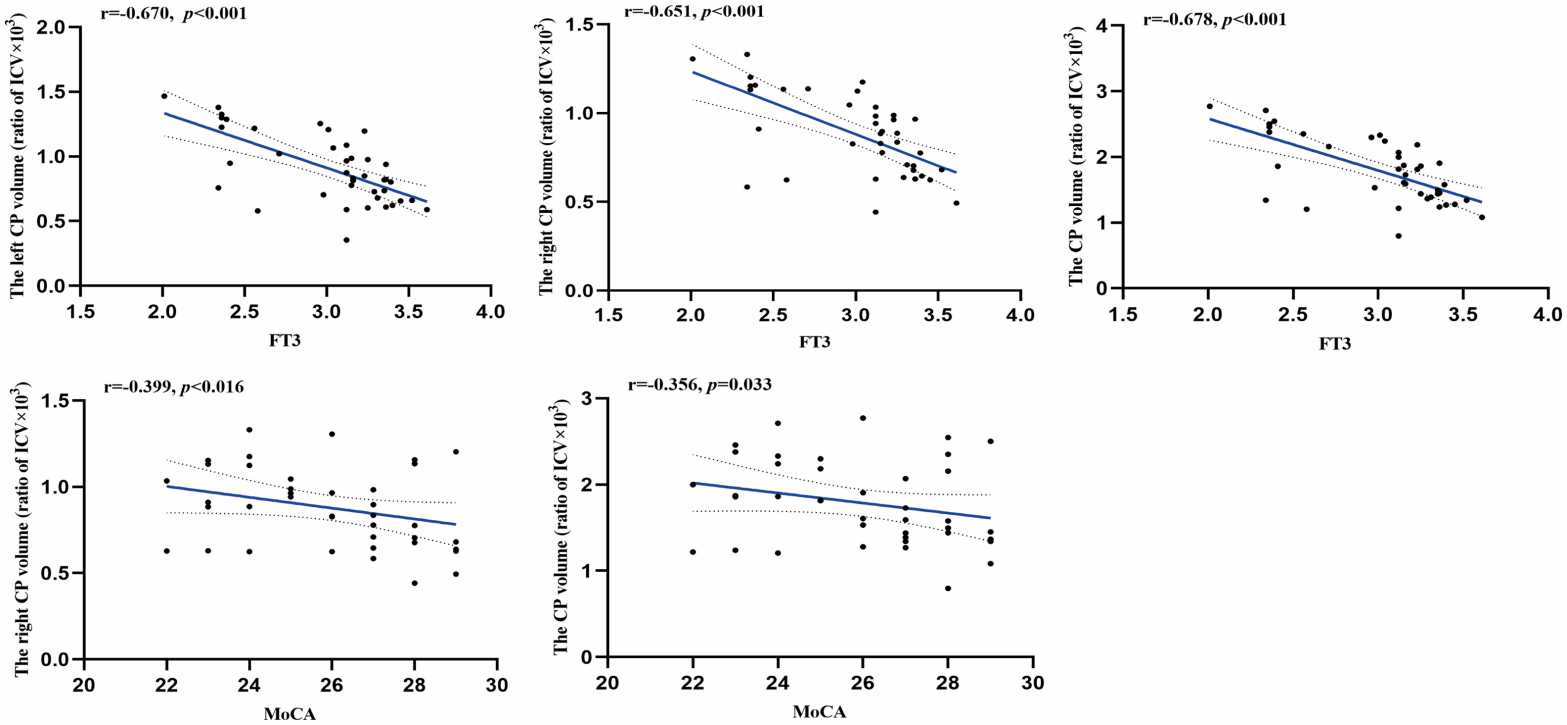
Relationships between choroid plexus (CP) and clinical variables as well as neuropsychological assessments. Correlation coefficients (*r*) and *p* values were obtained using partial correlation analysis, controlling for age, sex, body mass index, years of education, smoking status, and alcohol consumption. Trend lines depict linear regression with 95% confidence intervals (shaded). FT3, free triiodothyronine; MoCA, Montreal Cognitive Assessment.

As shown in Table 4 and Figure 6, after adjusting for age, sex, BMI, years of education, smoking, and alcohol consumption, FT3 levels were negatively correlated with absolute CP volumes in the left, right, and whole brain (*r* = −0.670, *p* < 0.001; *r* = −0.651, *p* < 0.001; *r* = −0.678, *p* < 0.001). Moreover, MoCA scores were negatively correlated with absolute CP volumes in the right hemisphere and whole brain (*r* = −0.399, *p* = 0.016; *r* = −0.356, *p* = 0.033). Overall, larger CP volumes were associated with lower FT3 levels and poorer cognitive performance. This directionality was consistent with the ALPS-related findings, suggesting that these two imaging markers may show convergent clinical relevance.

**Table 4:** Associations between clinical characteristics, neuropsychological assessments, and CP volume.

Variables	The left CP volume (ratio of TIV×10^3^)		The right CP volume (ratio of TIV×10^3^)		The CP volume (ratio of TIV×10^3^)	
	*r*	*p* value	*r*	*p* value	*r*	*p* value
Duration(Months)	0.183	0.284	0.276	0.103	0.231	0.175
FT3 (pmol/L)	−0.670	<0.001	−0.651	<0.001	−0.678	<0.001
FT4 (pmol/L)	−0.108	0.531	−0.052	0.762	−0.084	0.627
TSH(mIU/L)	0.113	0.511	0.030	0.863	0.076	0.660
TPOAb(IU/mL)	−0.048	0.781	−0.072	0.677	−0.060	0.727
TgAb(IU/mL)	0.109	0.528	0.053	0.759	0.085	0.624
MoCA	−0.310	0.065	−0.399	0.016	−0.356	0.033
HAMD	−0.194	0.256	−0.245	0.150	−0.222	0.192
HAMA	−0.349	0.057	−0.312	0.064	−0.339	0.053

Partial correlation analysis was performed to control for the confounding effects of age, sex, body mass index, years of education, smoking, and drinking. Trend lines depict linear regression with 95% confidence intervals (shaded). CP, choroid plexus; TIV, total intracranial volume; FT3, free triiodothyronine; FT4, free thyroxine; TgAb, anti-thyroglobulin antibodies; TPOAb, thyroid peroxidase antibodies. Bold indicates statistical significance; MoCA, Montreal Cognitive Assessment; HAMD-24, Hamilton Rating Scale for Depression-24; HAMA, Hamilton Anxiety Rating Scale.

## 4 Discussion

This study is the first to investigate the role of the glymphatic system in OH. Using multiparametric MRI, we systematically evaluated glymphatic function in patients with OH and explored its associations with clinical characteristics and neuropsychological measures. Our findings revealed that patients with OH exhibit enlarged CP volumes, which indicate increased CSF production, alongside reduced DTI-ALPS index, reflecting impaired glymphatic activity. Moreover, these alterations were significantly correlated with clinical indicators and neuropsychological scores. Together, these results provide compelling evidence that glymphatic function, encompassing both CSF production/secretion and glymphatic circulation, is markedly disrupted in OH.

### 4.1 Decreased DTI-ALPS index in OH

The glymphatic system serves as a crucial pathway for the clearance of metabolic waste products from the brain. Its function can be influenced by multiple physiological factors, including arterial pulsation, sleep disturbances, body posture, and aging (33, 34). In the present study, patients with OH exhibited a consistent reduction in the DTI-ALPS index, suggesting impaired perivascular/interstitial fluid dynamics related to glymphatic function. Thyroid hormones are implicated in regulating astrocytic function and activation in the central nervous system (35), and astroglial involvement has been proposed as a mechanistic link between thyroid dysfunction and neuropsychiatric manifestations (36). Accordingly, thyroid hormone deficiency may plausibly affect astroglial water-transport–dependent perivascular exchange (e.g., AQP4-related processes), which could contribute to reduced ALPS-derived measures (17). Although direct evidence in adult OH remains limited, it is plausible that reduced T3 availability in OH may contribute to altered AQP4 expression or polarization, thereby affecting glymphatic fluid transport. In addition, arterial pulsatility provides an important driving force for CSF influx and glymphatic flow (37, 38). In addition, neuroinflammatory activation and astrocytic reactivity could further disturb perivascular CSF–interstitial exchange and clearance efficiency, providing another plausible pathway for a lower ALPS index. Prior studies have reported reduced cerebral blood flow in OH (39), which may indicate diminished vascular driving forces for perivascular fluid movement. This concept is consistent with observations in aging models, where reduced arterial pulsatility is accompanied by impaired glymphatic clearance efficiency (40). Because arterial pulsatility was not directly assessed in the present study, this interpretation remains speculative and is based on prior literature. Taken together, astroglial-related alterations and cerebrovascular hypoperfusion may represent complementary pathways through which OH could contribute to reduced ALPS-derived glymphatic-related measures. Consistent with our findings in OH, prior studies in AD and Parkinson’s disease have reported reduced DTI-ALPS index (41–44). Together, these data suggest a cross-disease pattern of glymphatic-related impairment. Notably, unlike primary neurodegenerative disorders, OH represents an endocrine–metabolic context in which ALPS reduction co-occurs with CP enlargement and thyroid-hormone–related associations, indicating potentially distinct upstream drivers.

### 4.2 Increased CP volume in OH

Beyond perivascular transport, glymphatic function is also closely related to CSF production and turnover. The CP, as a key structure responsible for CSF production and a major component of the blood-CSF barrier, may influence glymphatic circulation by modulating CSF supply and broader neuroimmune homeostasis. In our study, patients with OH showed bilaterally increased CP volumes, indicating a global CP structural alteration. Prior work suggests that CP enlargement can be observed in neurodegenerative disorders and may relate to altered CSF turnover and barrier/immune functions (45–47). In OH, chronic low-grade inflammation activity and metabolic slowing may promote CP epithelial–stromal remodeling and blood–CSF barrier alterations, which could contribute to CP enlargement and potentially affect CSF turnover. Although CP volume is not a direct measure of CSF production, volumetric changes may reflect CP tissue remodeling that could be relevant to CSF dynamics and glymphatic-related clearance.

### 4.3 Inverse association between DTI-ALPS index and CP volume

Importantly, we observed a significant inverse relationship between CP volume and the DTI-ALPS index, implying that individuals with larger CP volumes tended to exhibit lower ALPS index. One plausible interpretation is that CP structural remodeling and perivascular transport impairment may represent coupled components within a shared CSF–perivascular clearance framework. Previous studies have suggested that CP enlargement and glymphatic dysfunction may co-occur and contribute to the accumulation of pathological substrates in neurodegenerative diseases (45, 46). CP enlargement has also been associated with reduced CSF turnover, and volumetric changes in the CP may indirectly reflect glymphatic function (47). Alternatively, CP enlargement may reflect compensatory or inflammatory remodeling in response to impaired clearance or altered CSF turnover, which could coincide with reduced perivascular exchange. Notably, the negative association between FT3 levels and CP volume in our data further suggests that CP structural changes may track thyroid functional status in OH, supporting CP volume as a potentially sensitive imaging phenotype related to disease severity. From a methodological perspective, the interpretability of DTI-ALPS is supported by prior validation studies. For example, Zhang et al. (24) demonstrated an association between the DTI-ALPS index and glymphatic clearance assessed using intrathecal contrast administration. In addition, Taoka et al. (48) reported that when acquisition and post-processing are standardized, ALPS-derived measures show reasonable stability and comparability across scanners. Collectively, these data support the use of DTI-ALPS and CP volume as complementary markers capturing distinct but related aspects of glymphatic physiology in OH.

### 4.4 Clinical relevance: associations with clinical variables, and neuropsychological measures

Clinically, we found that the DTI-ALPS index was positively correlated with MoCA scores, whereas CP volume was negatively correlated with MoCA scores in patients with OH. These convergent associations suggest that glymphatic-related dysfunction and CP structural alteration may be linked to cognitive vulnerability in OH. This pattern aligns with previous reports that glymphatic dysfunction is associated with cognitive impairment (19, 49). Reduced DTI-ALPS index have been reported to correlate with cognitive decline (41, 42), and enlarged CP volume has also been proposed as an imaging correlate of cognitive deterioration (50, 51). Together, our findings extend this literature by suggesting that, in OH, both perivascular transport (ALPS) and CSF-related structural phenotypes (CP volume) are associated with cognitive performance. These results support the potential utility of the DTI-ALPS index and CP volume as noninvasive imaging markers for characterizing glymphatic-related alterations and identifying individuals at higher risk of cognitive impairment in OH, which may provide a useful perspective for early recognition and intervention.

This study has several limitations. First, as a cross-sectional study with a modest sample size, the findings should be interpreted cautiously, and longitudinal studies are needed to verify dynamic changes in the DTI-ALPS index and CP volumes. Second, although the DTI-ALPS index has been widely used as an imaging marker of glymphatic-related function, it remains an indirect measure rather than a direct assessment of glymphatic flow or clearance. Therefore, its physiological interpretation should be made with caution. The ALPS index may also be influenced by non-glymphatic factors, including white matter microstructural alterations, partial-volume effects in periventricular regions, and methodological variations in ROI placement and post-processing. Further validation using more direct glymphatic assessment approaches (e.g., contrast-enhanced MRI when feasible and ethically appropriate) and multimodal evidence is needed. Third, although measurements were conducted by a single investigator, the use of predefined anatomical coordinates and standardized pipelines minimizes observer bias. In addition, glymphatic function may be affected by cerebral perfusion, arterial pulsatility, sleep, or cardiovascular and physiological factors. Although we excluded participants with severe cardiovascular diseases to minimize confounding, these factors should be considered in future research.

This study is the first to systematically demonstrate characteristic alterations in patients with OH, including reduced DTI-ALPS index and CP volumes. Both measures were significantly associated not only with thyroid hormone levels but also with cognitive performance. These findings provide novel insights into the mechanisms underlying cognitive impairment in OH. Future longitudinal and interventional studies are warranted to investigate whether thyroid hormone replacement therapy can reverse glymphatic dysfunction and improve cognitive outcomes.

## Data Availability

The raw data supporting the conclusions of this article will be made available by the authors, without undue reservation.

## Footnotes

1
https://stnava.github.io/ANTs/

2
https://fsl.fmrib.ox.ac.uk/fsl/

3
https://github.com/Deep-MI/FastSurfer
